# Supplementary material for: emiRIT: a text-mining-based resource for microRNA information
Source: Database (Oxford). 2021 May 28;2021:baab031. doi: 10.1093/database/baab031 (PMC8163238; doi:10.1093/database/baab031)
Supplement: baab031_Supp [file baab031_supp.zip › SupplementaryTable_S1...docx]

| Type | Regular Expression | miRNA Example |
| --- | --- | --- |
| 1 | (((?<=[^a-zA-Z])\|^)[a-zA-Z]{1,3}-?)?(microR\|\(miR\)\|miRNA\|micro( \|-)?RNA\|miRN\|miR\|mir)s?(-\|_\|x\| )?([0-9]+([a-g\*p]+[0-9]*)?) | hsa-miR-21,  miR-34a,  microRNA-199-5p,  microRNA-199-3p |
| 2 | (((?<=[^a-zA-Z])\|^)[a-zA-Z]{1,3}-?)?(let\|Let)s?(-\|_\|x\| )?(7[a-g\*p]*)(?=[^0-9]\|$) | Let-7a |
| 3 | (((?<=[^a-zA-Z])\|^)[a-zA-Z]{1,3}-?)?(lin\|Lin)s?(-\|_\|x\| )?(4[a-g\*p]*)(?=[^0-9]\|$) | Lin-4 |

Table S1: Regular expressions to identify base patterns of miRNAs
