# Supplementary figures and images for: emiRIT: a text-mining-based resource for microRNA information
Source: Database (Oxford). 2021 May 28;2021:baab031. doi: 10.1093/database/baab031 (PMC8163238; doi:10.1093/database/baab031)

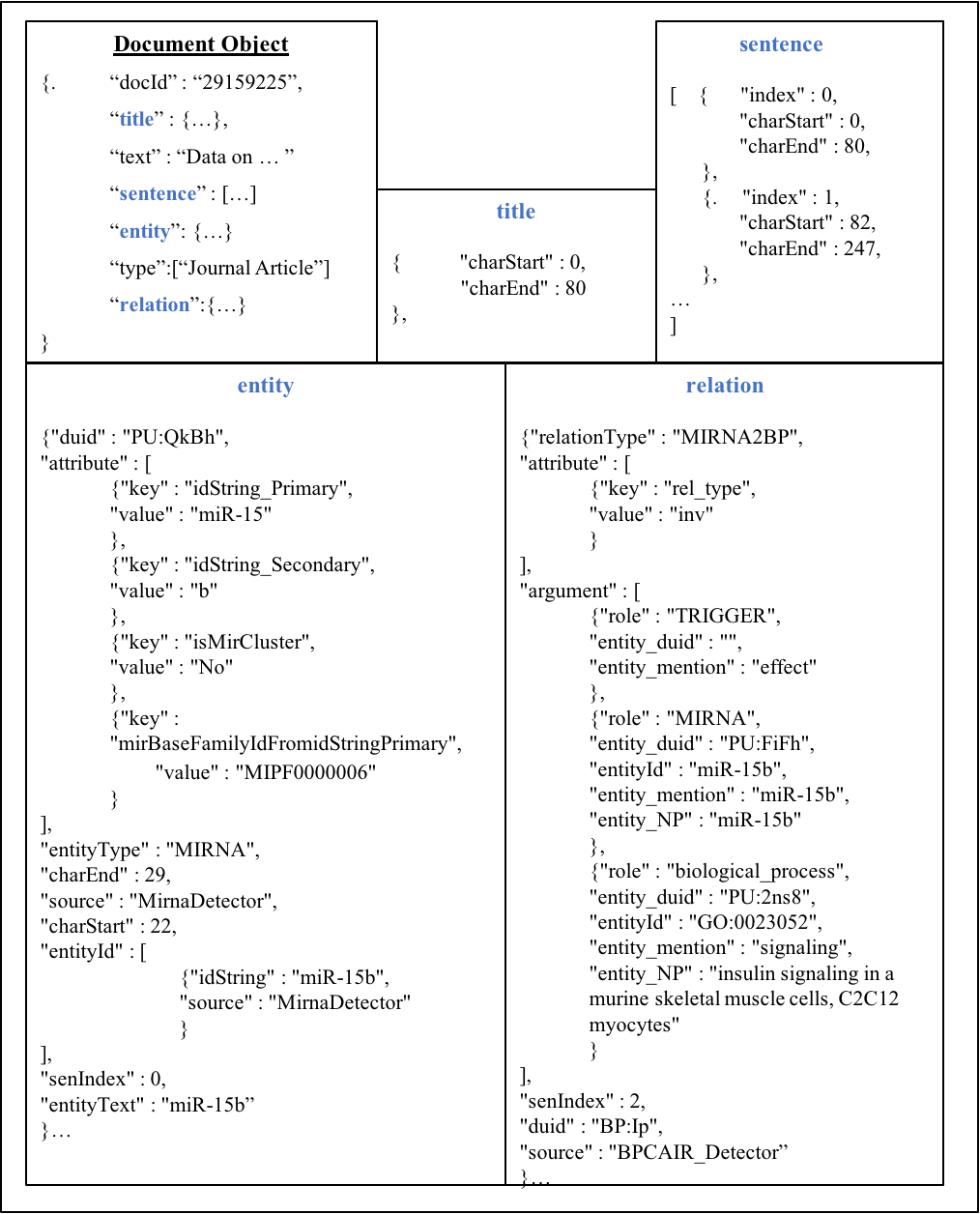


Figure S1: Overview of the JSON format of data stored in the emiRIT database

Supplement: baab031_Supp [file baab031_supp.zip › SupplementaryFigure_S1...docx]
